# Sensitivity of winter North Atlantic-European climate to resolved atmosphere and ocean dynamics

**DOI:** 10.1038/s41598-019-49865-9

**Published:** 2019-09-16

**Authors:** Reindert J. Haarsma, Javier García-Serrano, Chloé Prodhomme, Omar Bellprat, Paolo Davini, Sybren Drijfhout

**Affiliations:** 1 Koninklijk Nederlands Meteorologisch Instituut (KNMI), P.O. Box 201, 3730 AE De Bilt, The Netherlands; 2 Group of Meteorology, Universitat de Barcelona (UB), Barcelona, Spain; 3 Barcelona Supercomputing Center (BSC), Barcelona, Spain; 4 Istituto di Scienze dell’Atmosfera e del Clima (CNR-ISAC), Torino, Italy; 5 IMAU, University of Utrecht, Utrecht, The Netherlands; 6 School of Ocean and Earth sciences, NOC Southampton, University of Southampton, Southampton, UK

**Keywords:** Atmospheric dynamics, Physical oceanography

## Abstract

Northern Hemisphere western boundary currents, like the Gulf Stream, are key regions for cyclogenesis affecting large-scale atmospheric circulation. Recent observations and model simulations with high-temporal and -spatial resolution have provided evidence that the associated ocean fronts locally affect troposphere dynamics. A coherent view of how this affects the mean climate and its variability is, however, lacking. In particular the separate role of resolved ocean and atmosphere dynamics in shaping the atmospheric circulation is still largely unknown. Here we demonstrate for the first time, by using coupled seasonal forecast experiments at different resolutions, that resolving meso-scale oceanic variability in the Gulf Stream region strongly affects mid-latitude interannual atmospheric variability, including the North Atlantic Oscillation. Its impact on climatology, however, is minor. Increasing atmosphere resolution to meso-scale, on the other hand, strongly affects mean climate but moderately its variability. We also find that regional predictability relies on adequately resolving small-scale atmospheric processes, while resolving small-scale oceanic processes acts as an unpredictable source of noise, except for the North Atlantic storm-track where the forcing of the atmosphere translates into skillful predictions.

## Introduction

Recent studies with global high-resolution climate models, with resolutions less than 50 km and 0.25° for the atmosphere and ocean respectively, have demonstrated the added value of enhanced horizontal resolution compared to the output from CMIP5 models that have a typical resolution of 150 km and 1°^1–13^. Key regions that are sensitive to horizontal resolution are the western boundary currents, with sharp frontal structures in the ocean as well as in the atmosphere that are associated with intense ocean-atmosphere heat exchange^1–6^. They are located in the genesis regions of baroclinic mid-latitude storms, such as the Gulf Stream-Newfoundland area – the target of this study. Sea surface temperature (SST) gradients there are communicated to the entire free troposphere, by the induced surface wind convergence, resulting in precipitation bands and deep moist convection along the front^1,14^. Diabatic heating induced by these processes modulates the genesis and development of baroclinc distrurbances^15–17^, which due to non-linear energy transfer also modifies larger planetary structures such as storm-track and blockings^12^. Recent research has highlighted the dynamics involved in this western boundary current, the air-sea interaction there and the sensitivity to model resolution^3,9^. In these studies the main focus was on near-term climate change^3^ or changes in mean climate^9^ (i.e. climatology). The impact on mid-latitude interannual variability remains up to now largely unknown. Using an unprecedented large data-set of 170 seasonal forecasts with the climate model EC-Earth^18^ at different atmosphere and ocean resolutions, we assess for the first time the relative importance of resolved atmospheric and oceanic processes on winter North Atlantic-European (NAE) climatology and variability, and the implied consequences for predictability.

## Experimental Set-Up

Seasonal forecasts for the boreal winter (December-February, DJF) were performed at three different resolutions^19^: SRes (standard resolution: T255-ORCA1), IRes (intermediate resolution: T255-ORCA0.25), and HRes (high resolution: T511-ORCA0.25). The configuration of the atmosphere model IFS – Integrated Forecast System – at T255 (T511) corresponds approximately to 80 km (40 km) horizontal resolution; whereas the ocean model NEMO – Nucleus for European Modelling of the Ocean – is set at the nominal horizontal resolution provided by ORCA configuration where ORCA1 (ORCA0.25) corresponds to ~110 km (~25 km). The forecasts consist of 10 members spanning the period 1993–2009, launched every November 1^st^. Further details on the simulations and analysis are given in the Methods section.

## Results

### Impact on climatology

**Figure 1:**
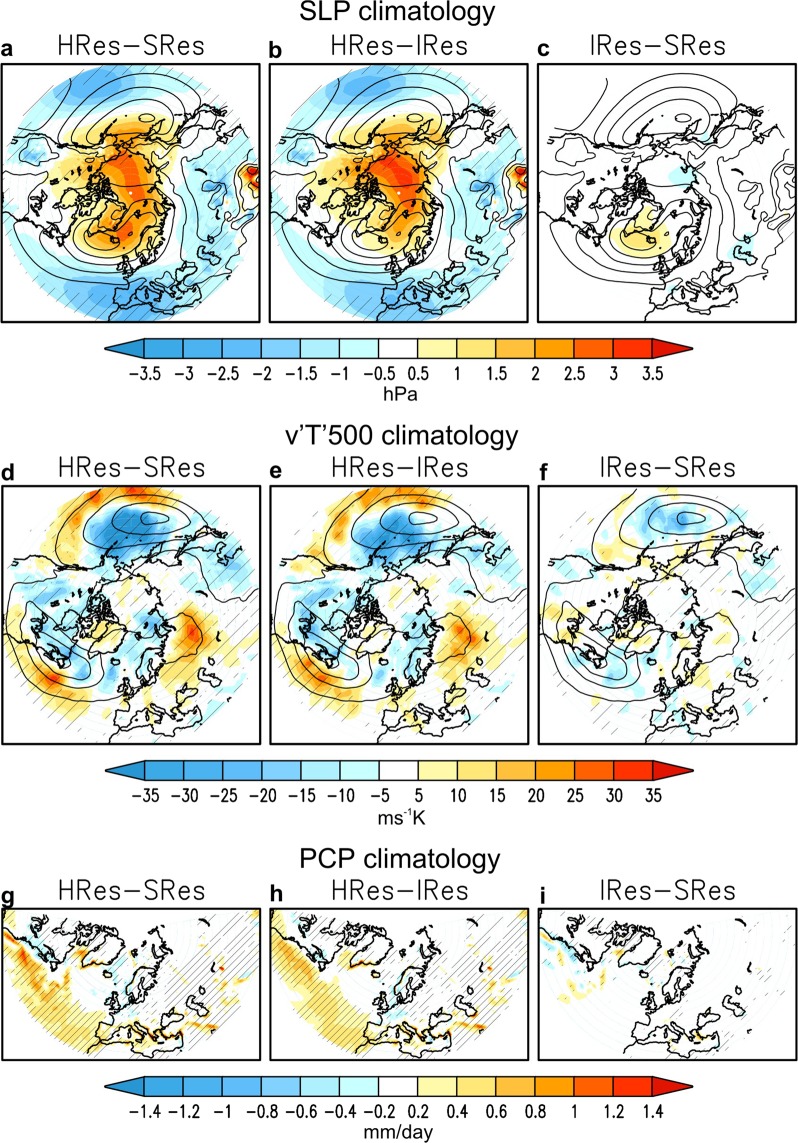
Sensitivity of DJF mean climate to model resolution. Upper panels (**a**–**c**): climatology (DJF) of SLP (hPa). Middle panels (**d**–**f**): climatology of v‘T’ (ms^−1^ K) at 500 hPa. Lower panels (**g**–**i**): climatology of precipitation (mm/day). Shading: HRes-SRes (**a**,**d**,**g**), HRes-IRes (**b**,**e**,**h**), IRes-SRes (**c**,**f**,**i**). Contours denote the SRes climatology of SLP (**a**–**c**) (interval 5 hPa, 1000–1020 hPa) and v‘T’ at 500 hPa (**d**–**f**) (interval 5 ms^−1^ K, 15–45 ms^−1^ K). Areas with the 95% confidence level are hatched.

**Figure 2:**
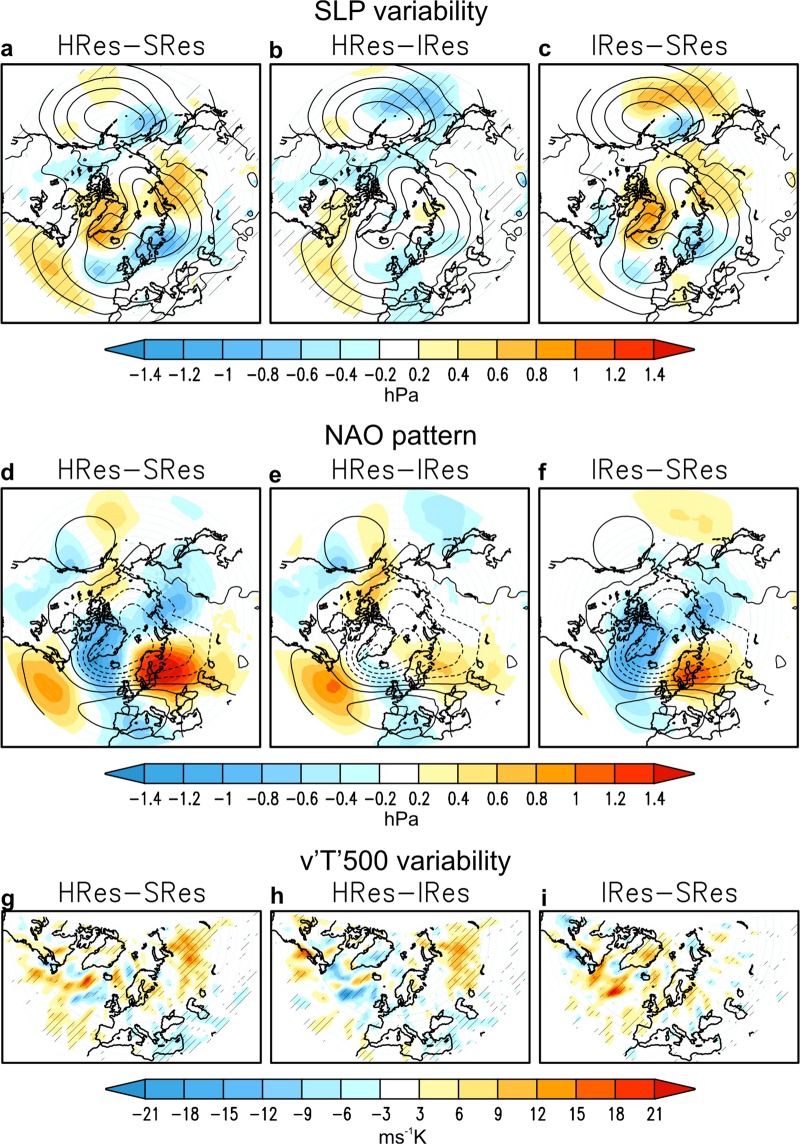
Sensitivity of DJF interannual variability to model resolution. Upper panels (**a**–**c**): interannual standard deviation of SLP (hPa). Shading: HRes-SRes (**a**), HRes-IRes (**b**) and IRes-SRes (**c**). Contours (interval 1 hPa, 2–5 hPa) denote SRes. Middle panels (**d**–**f**): SLP regression on the first empirical orthogonal function (EOF) of SLP over the North Atlantic (hPa). Shading: HRes-SRes (**d**), HRes-IRes (**e**) and IRes-SRes (**f**). Contours (interval 1 hPa, negative values dashed) denote SRes. The spatial patterns are scaled so that the values correspond to one standard deviation. Lower panels (**g**–**i**): interannual standard deviation of v‘T’ (ms^−1^ K) at 500 hPa. HRes-SRes (**g**), HRes-IRes (**h**) and IRes-SRes (**i**). Areas with the 95% confidence level are hatched.

In the NAE region, the climatological mean of winter sea level pressure (SLP) shows two well-known, semi-permanent pressure systems: the Azores High at middle latitudes and the Icelandic Low at subpolar latitudes. In its standard configuration (SRes; contours in Fig. 1-top), EC-Earth overestimates both centres of action by 1–2 hPa approximately; although stronger biases are found over other key semi-permanent pressure systems, where the model underestimates the Aleutian Low and overestimates the Siberian High by about 4–6 hPa (see Fig. S1). Increasing model resolution (HRes-SRes; Fig. 1a) has a significant impact on SLP climatology, particularly at mid-latitudes and over the polar cap. This translates into a reduction in the model bias in those areas (Fig. S1), implying a positive impact. Increasing only atmosphere resolution (HRes-IRes; Fig. 1b) provides a similar effect, implying that most of the signal can be attributed to atmospheric grid refinement. The lack of statistical significance in the SLP changes at subpolar latitudes is likely due to the high variability there (see contours in Fig. 2-top). On the other hand, increasing ocean resolution (IRes-SRes; Fig. 1c) barely affects the mean surface climate in the extratropics, including precipitation in the NAE region (Fig. 1-bottom), which is partly due to the ocean’s inability to respond at seasonal time-scales due to its large inertia.

The impact of increasing atmosphere resolution on the winter SLP climatology consists in a reduction of the meridional gradient that projects regionally on a negative North Atlantic Oscillation (NAO^20^)-like pattern, and hemispherically on a negative Arctic Oscillation (AO^20^)-like pattern. The effect on the meridional gradient of SLP (Fig. 1a,b) is associated with a southward displacement of the eddy activity in the cyclogenesis regions, here illustrated with changes in the transient-eddy heat flux at 500hPa (v‘T’; Fig. 1d,e). Such southward shift is present over the three main regions of eddy activity in the Northern Hemisphere, namely the North Pacific and North Atlantic storm-tracks and the low-pressure belt over the Ural-Siberian coast^20^. These results confirm the sensitivity of the climatological distribution of baroclinic disturbances, and thereby of the eddy-driven mean-flow, to atmosphere resolution that was earlier suggested from atmosphere-only simulations^21,22^. Because of the importance of properly representing storm-track dynamics in future-climate projections^23,24^, these results also envisage relevant outcomes from mesoscale-resolving atmosphere models in HighResMIP^25^.

### Impact on variability

The relative strength of the Azores High and the Icelandic Low is tightly linked to the NAO, which is the leading mode of regional atmospheric variability and strongly influences the interannual variability of surface temperature and precipitation as it is associated with the modulation of the westerly flow reaching the continent from the ocean and the North Atlantic storm-track^26^. Thus, assessing the relative impact of atmosphere and ocean resolution on the winter NAE circulation variability is as important as that on the circulation climatology. As introduced above, while the latter has been analysed in previous studies, the former is for the first time explored here.

Increasing ocean resolution (IRes-SRes; Fig. 2c) has a significant impact on SLP interannual variability, showing a longitudinal shift at subpolar latitudes with a marked increase of variability over Greenland, whereas increasing atmosphere resolution mainly results in a local enhancement of mid-latitude variability (HRes-IRes; Fig. 2b). The contributions appear to add linearly (HRes-SRes; Fig. 2a) and translate into changes in the spatial pattern of the NAO (Fig. 2d): increasing ocean resolution shifts the location of the northern centre of action (Fig. 2f), whereas increasing atmosphere resolution mainly affects the amplitude in the western part of the basin (Fig. 2e). Note that longitudinal shifts in the NAO pattern have important regional environmental significance^27–29^.

Although the model bias in the NAO pattern itself does not change substantially with model resolution (Fig. S2), understanding the changes in SLP variability is fundamental to gain insight into climate dynamics and the predictability of the system. Analysing interannual variability in v‘T’ (cf. with climatology in Fig. 1-middle) reveals that increasing ocean resolution induces more injection of wave activity downstream along the storm-track (Fig. 2i), whereas increasing atmosphere resolution enhances baroclinicity in the cyclogenesis region (Fig. 2h). The latter is consistent with results in the previous section on the better representation of meso-scale processes by only increasing atmosphere resolution^23,24^. The mechanism for the former needs more analysis, as follows.

**Figure 3:**
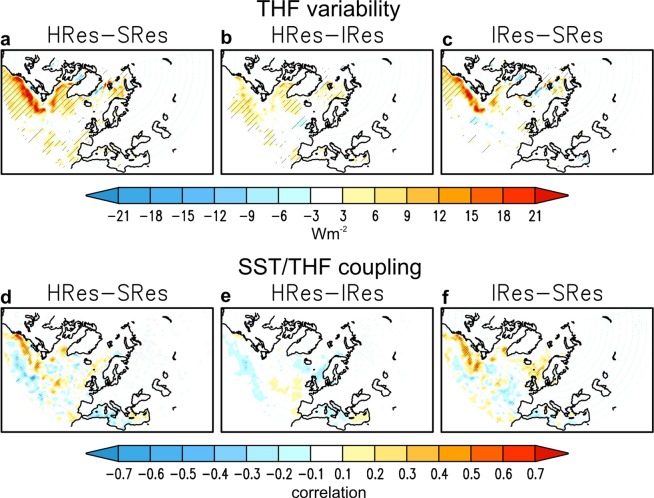
Sensitivity of DJF air-sea interaction variability to model resolution. Upper panels (**a–c**): interannual standard deviation of turbulent (latent plus sensible) heat flux (Wm^−2^). HRes-SRes (**a**), HRes-IRes (**b**) and IRes-SRes (**c**). Lower panels (**d**–**f**): interannual correlation between turbulent heat flux and SST. HRes-SRes (**d**), HRes-IRes (**e**) and IRes-SRes (**f**). Areas with the 95% confidence level are hatched.

The impact of increasing ocean resolution on SLP (Fig. 2c) and v‘T’ (Fig. 2i) variability is associated with significant changes in the interannual variability of SST along the Gulf Stream (Fig. S3-top) and of sea ice concentration (SIC) around the sea-ice edge of the Labrador Sea (Fig. S3-bottom). These changes represent an increase in variability of the surface forcing, and lead to an increase in turbulent heat flux (THF, sensible plus latent) variability (Fig. 3c). Strikingly, these changes are also associated with a stronger air-sea coupling, here measured as a higher correlation between SST and THF anomalies in these two regions of the North Atlantic (Fig. 3f). Together, these findings provide evidence in a coupled framework of the benefit of oceanic eddy-permitting resolution to improve representation of the air-sea interaction^1^, and for the first time of the impact of this oceanic forcing on the regional atmospheric variability.

### Implications for predictability

Skill in seasonal prediction relies, predominantly, on the ability of the forecast system to simulate correctly the impact of the slowly evolving components of the climate system, such as ocean heat content and soil moisture, on the dynamics of the atmosphere^30,31^. Because of the non-linear energy cascade in the climate system and the small-scale processes that dominate the momentum and heat exchange among the different components, it has been assumed that increasing resolution will be beneficial for climate forecasting.

**Figure 4:**
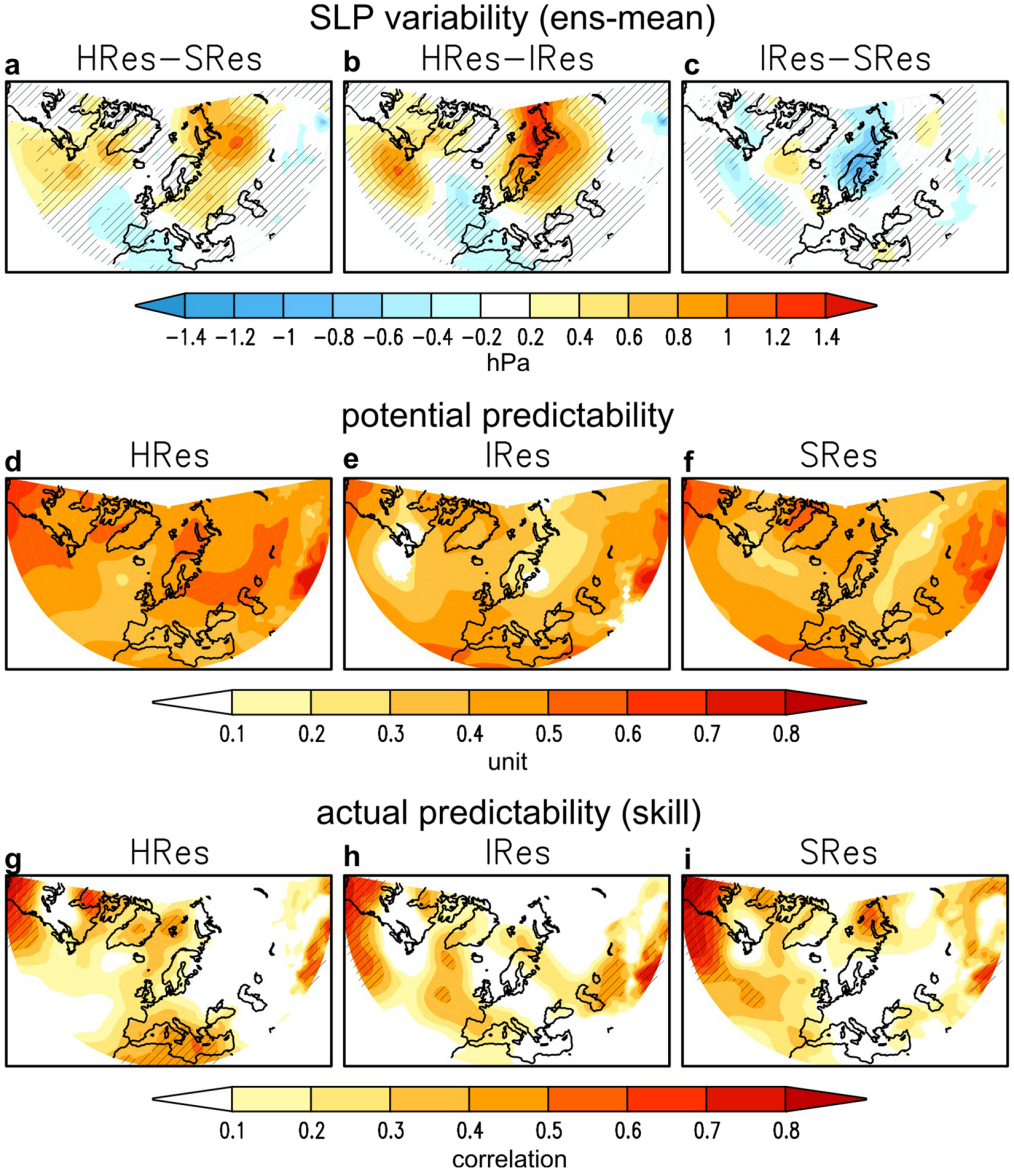
Sensitivity of DJF predictability to model resolution. Upper panels (**a**–**c**): σ_ens-mean_ (Methods) of SLP (hPa). HRes-SRes (**a**), HRes-IRes (**b**) and IRes-SRes (**c**). Middle panels (**d**–**e**): pp = σ_ens-mean_/σ_tot_ (Methods) of SLP. HRes (**d**), IRes (**e**) and SRes (**f**).Lower panels (**g**–**i**): Correlation of ensemble mean SLP with respect to ERA-Interim. HRes (**g**), IRes (**h**) and SRes (**i**). In the hatched area the correlation is significant at the 95% confidence level.

Increasing atmosphere resolution, indeed leads to an enhanced potential predictability (see Methods) over the western North Atlantic and northern Eurasia (Fig. 4d,e), which originates from an increased forced signal in those areas (Fig. 4b). Increasing ocean resolution, however, leads to a reduction in potential predictability in those areas (Fig. 4e,f) due to a decreased forced signal (Fig. 4c) and increased noise (Fig. 2c). The only region where increasing ocean resolution leads to an enhanced potential predictability is along the North Atlantic storm-track, east of Newfoundland (Fig. 4e,f), due to an increased forced signal (Fig. 4c), presumably linked to the reinforced wave injection (Fig. 2i), and reduced noise (Fig. 2c). Interestingly, this positive impact on potential predictability translates into statistically-significant actual predictability, namely prediction skill, in the northern North Atlantic (Fig. 4h). However, this increased potential predictability is destroyed by increasing atmosphere resolution (Fig. 4g) due to a reduction in the forced signal there (Fig. 4b). On the other hand, there is no direct translation of the enhanced potential predictability due to increasing atmosphere resolution over the western North Atlantic and continental Eurasia (Fig. 4b,d) into actual predictability (Fig. 4g). This implies that crucial processes in observations are not correctly represented by the model and/or better initializing the forecast system at high resolution might improve prediction skill.

## Summary and Conclusions

Using a hierarchy of coupled seasonal forecast experiments at different resolutions we have addressed the relative impact of resolving atmospheric and oceanic processes for regional climatology and variability. The influence of ocean dynamics on the Euro-Atlantic atmospheric variability and predictability is for the first time comprehensively discussed. It is shown that increased variability in SST along the Gulf Stream and SIC in the Labrador Sea – due to oceanic grid refinement – leads to significant changes in SLP interannual variability. Associated with the increase in turbulent heat flux variability, there is a stronger air-sea coupling in the North Atlantic and more injection of wave activity into the storm-track. Key in revealing this impact of ocean dynamics on atmospheric variability is the ability to generate mesoscale oceanic variability.

These results may well be model dependent, but represent the first comprehensive, statistically-significant evidence in a coupled system supporting the notion that increased ocean resolution, towards eddy-resolving, yields a stronger impact of the surface oceanic circulation on the atmospheric circulation. As no dedicated tuning was performed when increasing model resolution^19^, we suggest that further improvements may be achieved in the impacts reported here. Likewise, we encourage other modelling teams to follow a similar hierarchical approach in order to assess model diversity and help drawing model-independent conclusions. This may also include the intermediate step SRes-ocean/HRes-atmosphere, which was not available here, in order to fully assess possible non-linearities.

The enhanced variability associated with representing mesoscale oceanic perturbations overall results in reduced predictability, with the exception of the North Atlantic storm-track region that is presumably linked to enhanced wave injection and reduced noise. Increased predictability in the Euro-Atlantic sector is predominantly obtained by increasing atmosphere resolution that enables better capturing the signal that resides in the memory of the ocean. We note that there is a large scope for improvement in seasonal climate forecasting since most of this predictability does not yet lead to prediction skill.

Finally, while this study is focused on the North Atlantic-European climate, our results are also applicable to other regions characterized by a western boundary current such as the Kuroshio-Oyashio Extension^10,32^. As shown above, increasing atmosphere resolution has a dominant impact on the North Pacific atmospheric climatology (Fig. 1b,e). And, increasing ocean resolution strongly affects SLP variability along the North Pacific storm-track (Fig. 2c); however, this is counteracted by the effect of increasing atmosphere resolution (Fig. 2b), which results in the regional sensitivity of atmospheric variability to model resolution remaining unchanged (Fig. 2a) as opposed to the North Atlantic. Further exploring this discrepancy, however, is out of scope of this manuscript. In addition, no evidence has been found of remote effects from tropical SSTs on the sensitivity of the Euro-Atlantic atmospheric circulation to model resolution.

## Methods

The climate model is EC-Earth v3.0.1, which is an update of an earlier (v2.3) version^18^. The main new features are an improved radiation scheme^33^ and a new cloud microphysics scheme^34^. The climatology and variability of EC-Earth compares favourably with other GCMs^35–39^ illustrating the benefits of a climate model being derived from a weather model.

Retrospective seasonal forecasts were carried out over the period 1993–2009, with start dates every November 1st and for a forecast period of 4 months. For each start date 10 members were generated. The seasonal forecasts were performed at three different resolutions: SRes (standard resolution: T255-ORCA1), IRes (intermediate resolution: T255-ORCA0.25), and HRes (high resolution: T511-ORCA0.25). All simulations have L91 atmospheric configuration (91 vertical levels with top at 0.01hPa); ORCA1 and ORCA0.25 have L46 (46 vertical levels) and L75 (75 vertical levels) configuration respectively. The initial conditions were taken from ERA-Interim^40^ and GLORYS2v1^41^ for the atmosphere and ocean, respectively. The 10-member ensemble was generated by perturbing the atmosphere using singular vectors^42^. Further details of the simulations are described in^19^.

The analysis period is December-February (DJF) and the focus is the Northern Hemisphere mid-latitude climatology and variability; the analyses are therefore restricted to 20N–90N and later-on to the Euro-Atlantic sector. For the different fields analysed, the model climatology is computed as the ensemble-mean averaged across all start dates, whereas the model interannual variability is computed as the year-to-year differences in DJF anomalies (i.e. standard deviation) after linear detrending. A similar approach is followed for the atmospheric observational data, retrieved from the ERA-Interim reanalysis. Potential predictability (pp) is the predictability in a perfect model environment. It is defined here as pp = σ_ens-mean_/σ_tot_, where σ_ens-mean_ is the standard deviation of the ensemble-mean anomalies and σ_tot_ the standard deviation of all the members^43^. The time-mean covariance for transient-eddy heat flux, v‘T’ at 500 hPa^20^, is computed from filtered daily data using the 24 h filter^44^. The NAO is obtained as the leading Empirical Orthogonal Function of detrended SLP anomalies over 20N–90N/90W–40E. Statistical significance of differences in climatology (variability) is assessed with a two-tailed t-test (F-test) for equal means (variances) at 95% confidence level. Statistical significance of the prediction skill, with respect to ERA-Interim, is assessed with a one-tailed t-test for correlation at 95% confidence level, as only positive correlations indicate skill; note that negative correlations are masked out.

## Supplementary information

Supplementary Information
