# Supplementary material for: Sensitivity of winter North Atlantic-European climate to resolved atmosphere and ocean dynamics
Source: Sci Rep. 2019 Sep 16;9:13358. doi: 10.1038/s41598-019-49865-9 (PMC6746796; doi:10.1038/s41598-019-49865-9)
Supplement: Supplementary file 1 — Supplementary Information [file 41598_2019_49865_MOESM1_ESM.docx]

**Supplementary information**

***Sensitivity of winter North Atlantic-European climate to resolved atmosphere and ocean dynamics***

Reindert J. Haarsma^1^, Javier García-Serrano^2,3^, Chloé Prodhomme^2,3^, Omar Bellprat^3^, Paolo Davini^4^, Sybren Drijfhout^1,5,6^

^1^ Koninklijk Nederlands Meteorologisch Instituut (KNMI), P.O. Box 201, 3730 AE De Bilt, The Netherlands.

^2^Group of Meteorology, Universitat de Barcelona (UB), Barcelona, Spain

^3^Barcelona Supercomputing Center (BSC), Barcelona, Spain

^4^Istituto di Scienze dell’Atmosfera e del Clima (CNR-ISAC), Torino, Italy

^5^IMAU, University of Utrecht, Netherlands

^6^Oceanographic department, University of Southampton, Southampton, UK.

rein.haarsma@knmi.nl

**
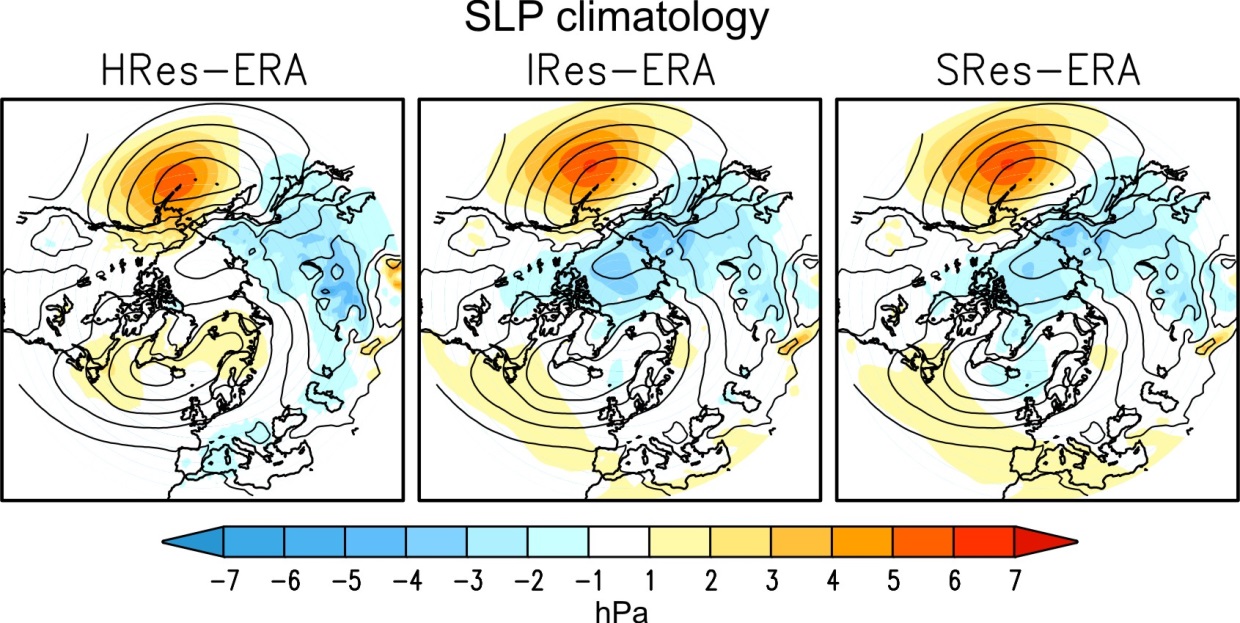
**

**Fig. S1** Shading: difference in SLP (hPa) climatology between ERA-Interim and HRes (left), IRes (middle), SRes (right). Contours (1000-1020 hPa, interval 5 hPa) in all panels denote SLP climatology of SRes.

**
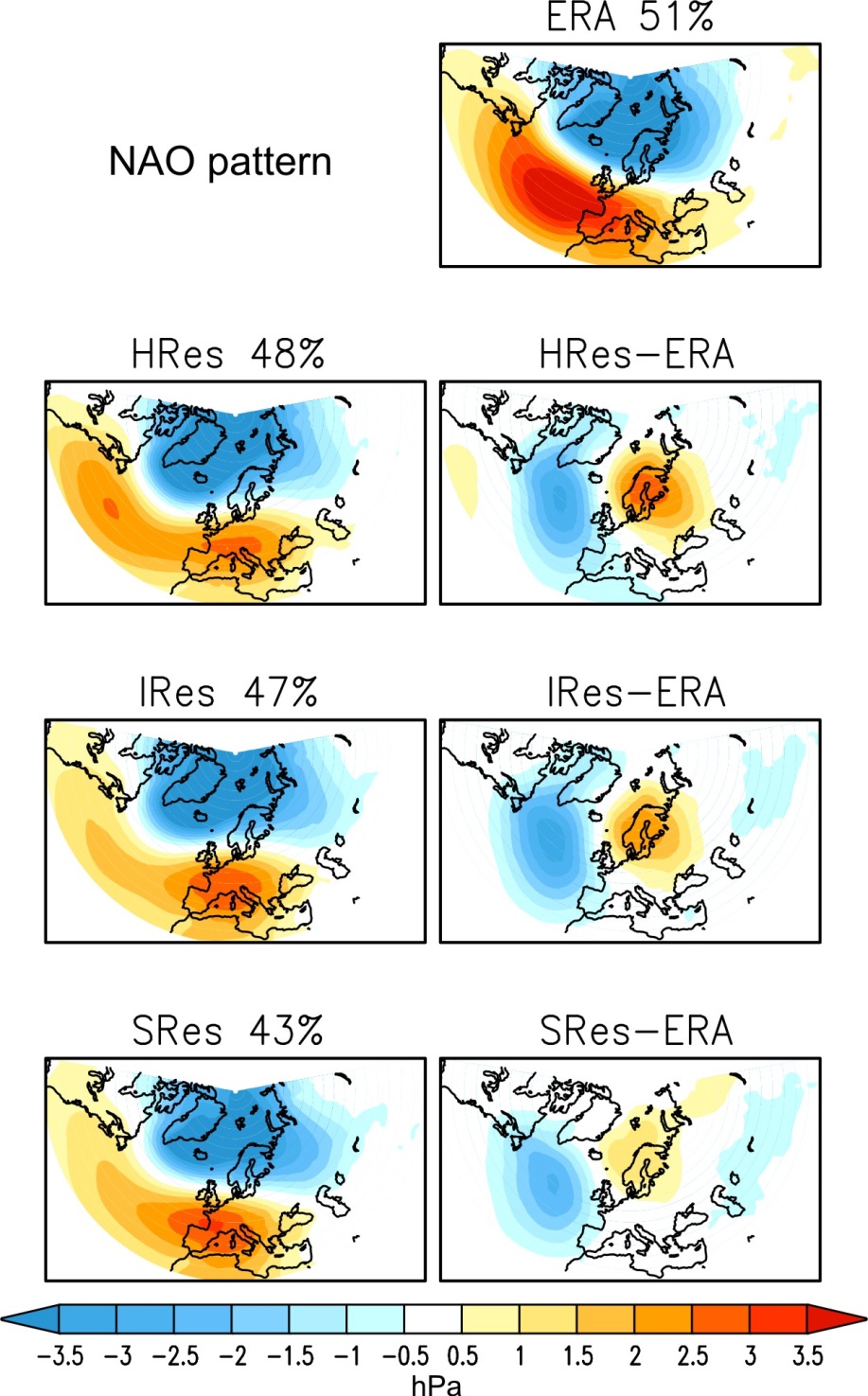
**

**Fig. S2** Upper panel: First empirical orthogonal function (EOF) SLP pattern of ERA-Interim. Second to fourth row: First EOF SLP patterns of HRes (2^nd^ row), IRes (3^rd^ row) and SRes(4^th^ row) and the difference with the first EOF SLP of ERA-Interim. The shading indicates hPa. The spatial patterns are scaled so that the values correspond to one standard deviation. The numbers on top of the panels denote the explained variance.

**
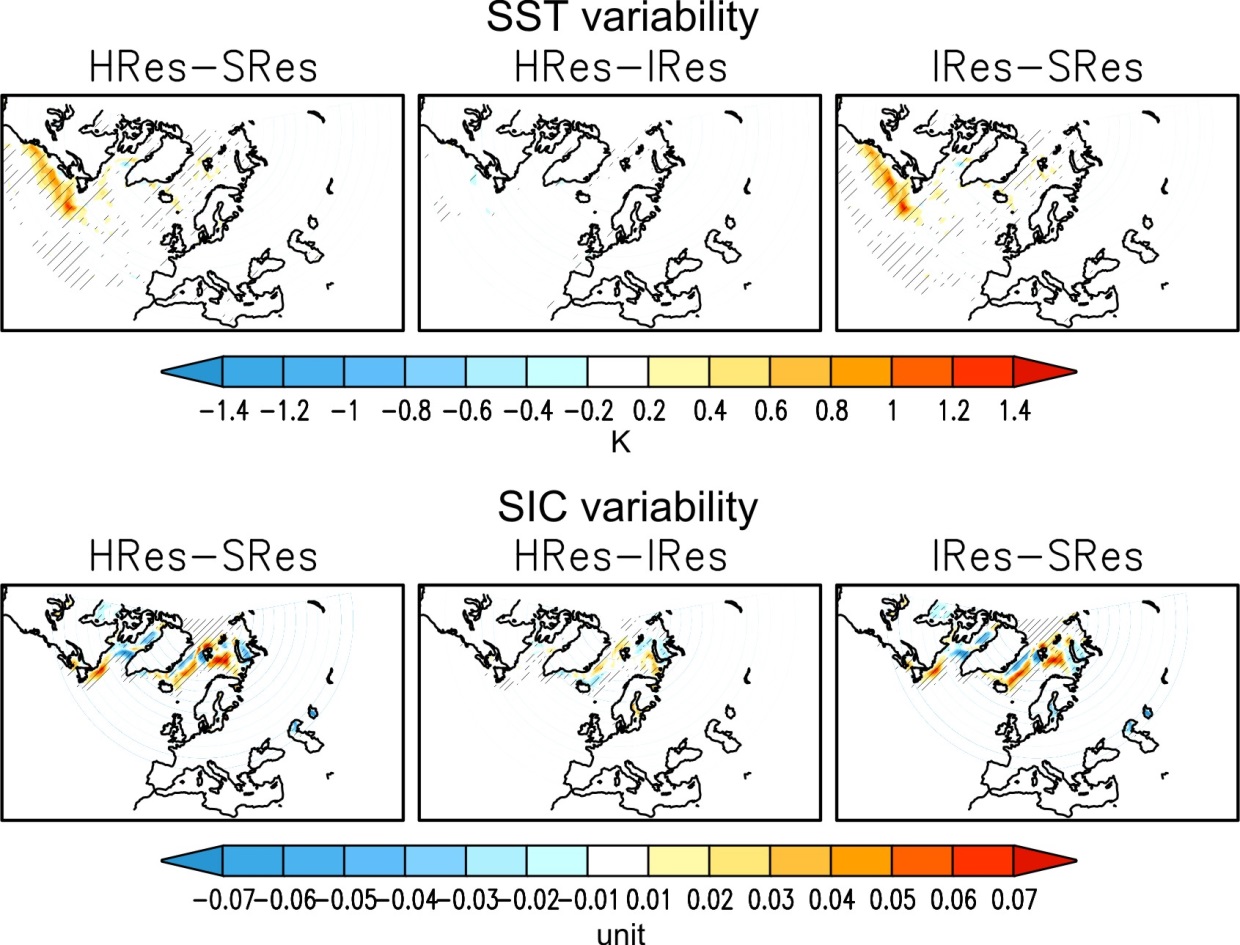
**

**Fig. S3** Upper panels: standard deviation of interannual seasonal mean (DJF) SST variability (K). Lower panels: standard deviation of interannual seasonal mean (DJF) variability of SIC (fraction).

**
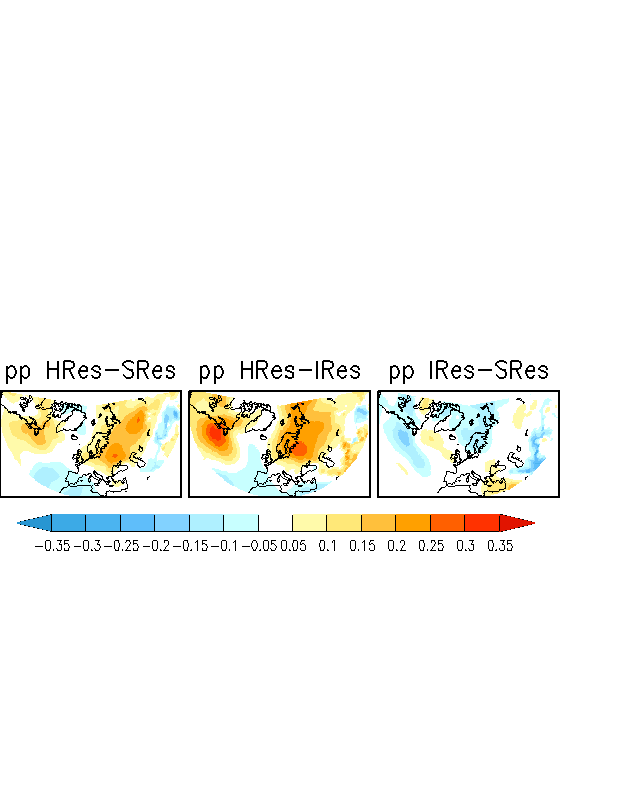
**

**Fig. S4** Difference in potential predictability (pp) (see Methods) between HRes and SRes (left), HRes and IRes (middle), IRes and SRes (right).
